# Potential of melatonin to reverse epigenetic aberrations in oral cancer: new findings

**DOI:** 10.17179/excli2023-6624

**Published:** 2023-12-12

**Authors:** Emilio Gil-Martín, Eva Ramos, Francisco López-Muñoz, Javier Egea, Alejandro Romero

**Affiliations:** 1 Department of Biochemistry, Genetics and Immunology, Faculty of Biology, University of Vigo, 36310 Vigo, Spain; 2 Department of Pharmacology and Toxicology, Faculty of Veterinary Medicine, Complutense University of Madrid, 28040 Madrid, Spain; 3 Faculty of Health, Camilo José Cela University of Madrid (UCJC), 28692 Madrid, Spain; 4 Neuropsychopharmacology Unit, Hospital 12 de Octubre Research Institute, 28041 Madrid, Spain; 5 Unidad de Investigación, Hospital Santa Cristina, Instituto de Investigación Sanitaria Princesa (IIS-IP), 28006 Madrid, Spain

**Keywords:** melatonin, oral cancer, epigenetic, gene expression, microRNAs, adjuvant therapy

## Abstract

It is now an accepted principle that epigenetic alterations cause cellular dyshomeostasis and functional changes, both of which are essential for the initiation and completion of the tumor cycle. Oral carcinogenesis is no exception in this regard, as most of the tumors in the different subsites of the oral cavity arise from the cross-reaction between (epi)genetic inheritance and the huge challenge of environmental stressors. Currently, the biochemical machinery is put at the service of the tumor program, halting the cell cycle, triggering uncontrolled proliferation, driving angiogenesis and resistance to apoptosis, until the archetypes of the tumor phenotype are reached. Melatonin has the ability to dynamically affect the epigenetic code. It has become accepted that melatonin can reverse (epi)genetic aberrations present in oral and other cancers, suggesting the possibility of enhancing the oncostatic capacity of standard multimodal treatments by incorporating this indolamine as an adjuvant. First steps in this direction confirm the potential of melatonin as a countermeasure to mitigate the detrimental side effects of conventional first-line radiochemotherapy. This single effect could produce synergies of extraordinary clinical importance, allowing doses to be increased and treatments not to be interrupted, ultimately improving patients' quality of life and prognosis. Motivated by the urgency of improving the medical management of oral cancer, many authors advocate moving from *in vitro* and preclinical research, where the bulk of melatonin cancer research is concentrated, to systematic randomized clinical trials on large cohorts. Recognizing the challenge to improve the clinical management of cancer, our motivation is to encourage comprehensive and robust research to reveal the clinical potential of melatonin in oral cancer control. To improve the outcome and quality of life of patients with oral cancer, here we provide the latest evidence of the oncolytic activity that melatonin can achieve by manipulating epigenetic patterns in oronasopharyngeal tissue.

## Introduction

Heterogeneous primary malignancies arising from the epithelial lining of the lips, mouth, hypopharynx, oropharynx, and larynx are collectively termed oral (cavity) cancers because they share major risk factors and the first two histotypes account for ~85 % of the global incidence. In addition, ~90 % of the tumor entities arising from this anatomical region are G2 squamous cell carcinomas, usually originating from the labial border, from where they spread aggressively (Johnson et al., 2020[80]; Miranda-Filho and Bray, 2020[127]) eventually accounting for ~75 % of head and neck squamous cell carcinomas (Chen et al., 2020[26]). Approximately twice as common in men than women, usually over the age of 60, although cases in young people are rising sharply (Bray et al., 2018[16]), oral cancer accounts for ~4.5 % of all tumors, with recent estimates predicting more than 870,000 diagnoses and more than 400,000 deaths worldwide by 2020 (Sung et al., 2021[193]).

Despite the evidence that a small proportion of oral (cavity) tumors show familial and/or ethnic clustering, the prevailing paradigm is that most patient-specific predisposition arises from cross-interactions between (epi)genetic substrates and damage caused by environmental factors (Lingen et al., 2011[101]). Specifically, serial (epi)genetic insults to the oronasopharyngeal epithelium by external aggressors exceed homeostatic adaptive thresholds and the cell is driven to a point of no return where atypia and dyshomeostasis take control. Thus, alcohol abuse and regularly smoked or smokeless tobacco have a major impact on almost 90 % of the prevalence, as they both directly and repeatedly expose the oronasopharyngeal mucosa to carcinogens and persistent inflammation (Irimie et al., 2018[75]; Pelucchi et al., 2006[150]). In Southeast Asian and Western Pacific countries, the etiological spectrum extends to pipe smoking, betel quid chewing containing areca nut, with or without tobacco, and nitrosamine-rich foods (Shield et al., 2017[181]). In addition, the new forms of smoking that are marketed as safe and are becoming popular worldwide, such as water pipes or electronic cigarettes, pose a new global health concern that is currently being researched due to the probable carcinogenicity of chemicals that encounter the epithelium (Chaturvedi et al., 2022[24]; Ebersole et al., 2020[40]; Patil et al., 2019[148]). Irrespective of the above-mentioned comorbidity factors, persistent human papillomavirus (HPV) infection, mainly HPV16 and HPV18 serotypes, has increased the carcinogenicity of the oropharynx, tonsils, and base of the tongue in males over the last two decades (Johnson et al., 2020[80]), mainly in high-income countries (Bosetti et al., 2020[13]; Lechner et al., 2022[91]). In this regard, epidemiological trends indicate that HPV is currently the cause of more than 80 % of oropharyngeal tumors, compared to its impact of ~2-3 % on overall incidence (Bouvard et al., 2022[15]; Chaturvedi et al., 2022[24]). Considering that aging, heavy alcohol intake, smoking, tobacco or betel nut chewing, and HPV infection are the main causes of oral carcinogenesis, tumors of this origin can be largely avoided by eliminating tobacco and alcohol abuse, along with prophylactic HPV vaccination (Chaturvedi et al., 2018[25]; Cohen et al., 2018[32]).

## Opportunities for Epigenetics in the Treatment of Oral Cancer

The definition of oral (cavity) cancer as a progressive and multifactorial disease (Lingen et al., 2011[101]) entails differentiated etiogenic pathways and histopathological subtypes (Bavle et al., 2016[11]), with specific characteristics and treatment needs. However, in the absence of stage- and tumor-specific biomarker-guided assessment, as in other cancers, both prognostic and therapeutic decisions are based on AJCC-TNM indexing. Morphological staging can occasionally lead to subjectivity and predictive discrepancies, particularly with regard to the prognosis of primary metastases, as evidenced by the different outcomes of small, low-risk non-metastatic, T1/T2 tumors (Wang et al., 2019[208]) or diffuse low-grade dysplasia (Crawford et al., 2022[33]). The reason for these discrepancies lies in the underlying complexity of the cytological/histological properties considered in the AJCC-TNM subclassification, such as microbiota imbalance (Pignatelli et al., 2022[154]), tumor microenvironment (Liu et al., 2022[103]) or perineural invasion (Hurnik et al., 2022[74]). Similarly, there is currently no standardized procedure to address the carcinogenic potential of silent (epi)genetic alterations that occur in tissues adjacent to the primary tumor (Strzelczyk et al., 2018[188]), which are thought to represent the reservoir of cancerization (oral field cancer) from which recurrence arises after removal and first-line treatment (Peralta-Mamani et al., 2022[152]). Therefore, to improve survival expectations, current cytohistopathological staging needs to evolve towards more precise stratification based on (epi)genetic signatures of the tumor and the microenvironment on which patient progression and treatment response depend. As a result, the ambitious Precision Medicine scenario of individualizing therapeutic decisions would be realistic (Li et al., 2020[92]; Makitie et al., 2022[120]; Sasahira et al., 2022[175]) and would enable us to avoid surgical disfigurement and functional disability (Chung et al., 2004[31]; Lingen et al., 2011[101]; Zhang et al., 2021[226]). In this regard, intensive research is attempting to develop a molecular-based staging system that provides reliable and non-invasive biomarkers to achieve early diagnostic protocols (Nijakowski et al., 2022[137]; Parfenova et al., 2021[146]; Santosh et al., 2016[174]).

Epigenetics refers to changes in DNA and chromatin architecture that, in close association and without altering the base sequence, can dictate how RNA activity and gene expression are modulated. The ensemble of chemical tags that play this regulatory role gives rise to the epigenome, a full member of the genetic information. The epigenetic profile comprises cell- and tissue-specific native traits that remain stable during cell division and are therefore mitotically and meiotically heritable. At the same time, epigenetic patterns are sensitive to the effects of endogenous and external stressors (oral habits, carcinogens, etc.) and, unlike genetic variants, are reversible and highly dynamic (Cavalli and Heard, 2019[22]). Notably, epigenetic plasticity is part of the homeostasic framework that underpins the resilience of cells and tissues. Furthermore, epigenetics has expanded the classical paradigm of gene-based disease and therapeutic practice, as epimutations, like genetic mutations, are potentially pathogenic and can be targeted to restore baseline configuration. The transient nature of the epigenetic landscape has also abolished the traditional segregation between genetic and environmental causes of the disease, as disordered epigenetic patterns crystallize the influence of non-genetic factors (Singh et al., 2016[184]). In addition, the reversibility of epigenetic alterations enables different approaches to the treatment of diseases, including cancer. Consequently, the epigenomic revolution has broadened the conceptualization of cancer pathways and epigenetic alterations have become part of the canonical hallmarks of cancer (Flavahan et al., 2017[50]). In this regard, oral carcinogenesis should be reinterpreted as the summation of (epi)mutations and progressive cellular damage from environmental stressors (Bais, 2019[9]).

**Figure 1:**
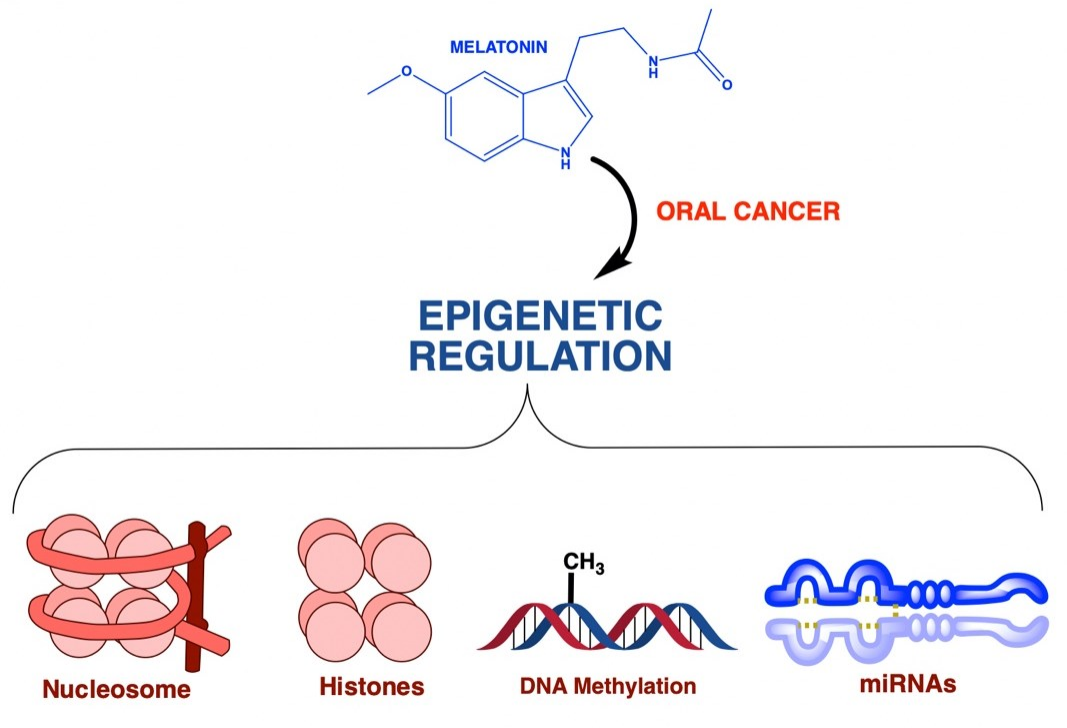
The role of melatonin in epigenetic regulation. By altering gene expression patterns without altering the DNA sequence itself, melatonin contributes significantly to the regulation of epigenetic processes in oral cancer. Many epigenetic processes are involved, including microRNA production, histone modification, and DNA methylation.

Genetic alterations leading to tumorization of the oronasopharyngeal structures are well known, with excellent investigations and comprehensive reviews published in recent years (Ali et al., 2017[7]; Usman et al., 2020[201]), which we will therefore not review here. This is not the case for epigenomic studies, which, although rapidly expanding, are still in the gap-filling phase to obtain as complete an epigenetic picture as possible of the different stages and locations of oral tumors. In cancer in general and in oral cancer in particular, the most studied epigenetic changes include gene silencing by DNA hypermethylation, induction of genome instability by DNA hypomethylation, post-translational modifications of chromatin histones (mainly, acetylation/ deacetylation and methylation/demethylation) and transcriptional regulation by small non-coding RNAs (especially micro-RNAs or miRs) (Figure 1(Fig. 1)). 

From a translational perspective, epigenetic research hopes to identify biomarkers for premalignant risk assessment, screening and early detection, differential diagnosis and stratification, recurrence/prognosis prediction and/or novel targeted therapies. Epimutations reshape the genome architecture and thus the degree of chromatin compaction, which facilitates or hinders DNA access and transcriptional regulation (Gazdzicka et al., 2020[54]). Based on the reversibility of epimutations, dynamic epigenotype restoration is conceptually plausible, although the inherent toxicity of some interventions makes achieving epigenetic normalization clinically unfeasible. Nevertheless, as with advances in other cancers, there is great promise in epigenetic pharmacology for the treatment of oral tumors. Pharmacology can contribute to the therapeutic armamentarium against oral cancer with epigenetically active compounds, especially the 'first generation' inhibitors of histone deacetylases (HDACi) and DNA methyltransferases (DNMTi), either as primary monotherapy or, more likely, as multi-drug regimens mixed with conventional treatments (Kumar et al., 2017[87]; Qi et al., 2016[155]). Combined treatments enhance oncolytic efficacy by acting synergistically on the same therapeutic target (signaling pathway, gene regulator, etc.) or simultaneously on different non-redundant critical targets. As evidence of their therapeutic efficacy, the US and European regulatory authorities have recently approved some combination regimens (Bondarev et al., 2021[12]; Majchrzak-Celinska et al., 2021[119]) to overcome drug resistance and improve the clinical management of oral cancer, which is currently based almost exclusively on conventional radiochemotherapy.

## Potential Contributions of Melatonin to the Carcinogenic Cycle and Treatment of Oral Cancer

Early oral cancer does not present with pain or significant somatic symptoms. The resulting delay in diagnosis allows for invasive spread to nearby lymph nodes and distant metastases, significantly worsening the prognosis of the disease (Guneri and Epstein, 2014[61]). Indeed, morbimortality has remained persistently high for decades across all age groups and tumor subsites, with ~60 % of cases diagnosed at advanced T3 and T4 stages. Specifically, 2-year recurrence and metastasis rates after resection reach 80 % (Chung et al., 2004[31]), and median 5-year survival rates for advanced cases are as low as ~10-50 % depending on stage and resectability, among the lowest of all common cancers (Wang et al., 2019[211]). However, early detection (stages T1 or T2) increases the 5-year survival rate to ~80 % (Ho et al., 2019[70]). The accessibility of the oral cavity, which facilitates examination and anamnesis, should improve detection, and avoid deep locoregional invasion or distant colonization. Disappointingly, clinicians do not have discriminative risk-predictive markers to differentiate benign oral lesions from those with serious malignant potential (Crawford et al., 2022[33]). In addition, standardized screening for massive or high-risk populations and secondary prevention strategies to reduce locally aggressive and/or metastasized lesions have not been implemented. Instead, the standard management of oral cancer is entirely based on post-diagnosis modalities that include surgery, concurrent post-operative chemoradiation, and targeted therapies (Day et al., 2003[37]). However, curative efficacy is far from optimal, as a large proportion of resected primary tumors usually recur within a few months of treatment initiation. It is hoped that minimally invasive or some non-invasive alternatives will soon be available to reduce the delay in diagnosis, relapse during follow-up, off-target toxicity, and innate or acquired resistance to radiochemotherapy (Dharmawardana et al., 2020[39]; Salaric et al., 2021[171]).

Given the need to strengthen the therapeutic arsenal against oral cancer, the natural methoxyindole melatonin has recently been repeatedly postulated as an adjuvant capable of increasing the efficacy of multimodal approaches and mitigating unwanted side effects, thus improving the well-being of patients (Capote-Moreno et al., 2019[20]; Sung et al., 2020[192]). In this regard, the gold standard for the treatment of oral squamous tumors is concurrent radiochemotherapy, with cisplatin-based combination regimens as the first-line option. However, this and other drugs are dose limited due to their acute and long-term nephrotoxicity (Hanigan and Devarajan, 2003[65]). In this regard, melatonin reduced the expression of renal biomarkers and significantly improved the cisplatin-induced histopathological renal damage in several strains of rats (Abdel-Rahman Mohamed et al., 2022[2]; Ali et al., 2020[6]). Melatonin has also been shown to be competent in protecting organs damaged by other chemotherapeutic drugs, as in the case of doxorubicin-associated cardiotoxicity (Maleki Dana et al., 2022[121]), which melatonin counteracted by preventing mitochondrial oxidative damage and cardiomyocyte ferroptosis (Sun et al., 2022[190]). On the other hand, the toxicity exerted *in vitro* by the pro-carcinogenic xenoestrogen bisphenol-A on human gingival fibroblasts, colon cancer and bone marrow stem cells was abolished by melatonin by reducing oxidative stress and genotoxicity (Ebrahimi et al., 2021[41]).

In terms of radiation-induced damage, melatonin can protect against hematological and intestinal lesions in mice exposed to systemic radiotherapy, significantly increasing their survival after daily doses of 100 mg/kg (Tripathi et al., 2022[198]). Inflammatory damage caused by ionizing radiation leads to severe mucositis in 90 % of patients undergoing irradiation (Brown and Gupta, 2020[17]). Without more satisfactory management than symptomatic treatment, mucositis forces the suspension of therapies and prolongs their duration, increasing costs, and compromising patient outcomes (Elting et al., 2007[43]). In this context, irradiation of the rat tongue has been reported to decrease internal melatonin, whereas topical application of a 3 % gel restored melatonin levels and protected the mucosa from ulceration by preventing mitochondrial disruption and NF-κB/NLRP3 inflammasome activation (Ortiz et al., 2015[142]). High content melatonin gels have also been shown to effectively counteract secondary oral (Abdel Moneim et al., 2017[1]) and intestinal (Fernandez-Gil et al., 2017[46]) mucositis in rat models subjected to tongue irradiation with or without conventional radiochemotherapy in oral cancer patients. Similarly, administration of melatonin (10 mg/kg/day), particularly prior to radiation, restored normal anti-inflammatory and antioxidant markers in rat liver (Yalcin et al., 2023[212]). Specifically, melatonin reduced mucositis and pain, avoiding treatment discontinuation and the need for morphine-dependent analgesia, thus paving the way for future phase III trials (Elsabagh et al., 2020[42]; Lozano et al., 2021[109]; Onseng et al., 2017[141]). However, when evaluating the efficacy of antioxidants in the treatment of mucositis, a recent meta-review of 13 randomized controlled trials found inconsistent results for melatonin in similar interventions (Raza et al., 2022[162]). Similarly, the systematic review and meta-analysis by Fan et al. (19 randomized controlled trials in various cancers) questioned the inclusion of melatonin in combination regimens as it does not appear to significantly improve relevant aspects of patient outcome such as quality of life, sleep regularization, fatigue, and pain (Fan et al., 2022[44]). In contrast to the claimed ineffectiveness of melatonin in reducing oral cancer mucositis and improving patient well-being, it should be noted that randomized, double-blind, placebo-controlled clinical trials have reported statistically significant benefits in delaying the onset of grade 3 oral stomatitis (Onseng et al., 2017[141]), pain reduction (Elsabagh et al., 2020[42]), and ulcer severity and duration (Lozano et al., 2021[109]). In addition, other recent systematic reviews and clinical trials have shown that melatonin co-administration improves inflammation (Moslehi et al., 2022[133]), ototoxicity (Basirat et al., 2023[10]), depression and ulceration (also reported in the Fan et al. study (2022[44])), as well as improved sleep/quality of life and reduced fatigue in patients with oral and other cancers (Jafari-Koulaee and Bagheri-Nesami, 2021[76]; Sedighi Pashaki et al., 2023[177]; Seo et al., 2023[178]; Zetner et al., 2023[223]). Therefore, most of the available evidence supports the suitability of melatonin formulations as an adjuvant to reduce mucositis and other side effects associated with radiochemotherapy regimens (González et al., 2019[56]), as well as to improve therapeutic efficacy and overcome drug resistance (Fernandez-Gil et al., 2019[47]; Ma et al., 2020[117]; Mafi et al., 2023[118]). Furthermore, clinical trials suggest that melatonin gels are highly penetrable and safe for the treatment of oral mucositis (Moslehi et al., 2022[133]). Thus, the issue to be resolved is not the usefulness of the indolamine, but rather the paucity of studies performed and their heterogeneity in many relevant parameters (size and type of cohort, treatment regimens, interventions, etc.), which probably contributes to the uncertainty and confusion regarding the advisability of including melatonin in the clinical management of patients with oral cancer.

Another important issue to be addressed is the recently reported pro-metastatic effect of long-term high-dose melatonin (5 mM *in vitro* for more than 10 generations) on oral squamous cell carcinoma cells, which progressed to malignancy and metastasis through pro-cancer upregulation of FGF19-dependent signaling through activation of the PER-eIFαATF4 stress-related pathway (Lang et al., 2021[89]). Conversely, 2 mM melatonin was shown to suppress the migration (24 h) and invasion (36 h) capabilities of the human tongue squamous cell carcinoma line SCC-15 via mRNA/protein downregulation of the pro-cancer FGF19/FGFR4 pathway (Wang et al., 2021[204]). In a recent systematic review by our research team, human equivalent doses of melatonin, extrapolated from those tested in animal studies, yielded significantly higher values (up to 24 times higher) than concentrations administered in randomized trials in breast cancer patients (Ramos et al., 2023[158]). Notably, regular doses of 20 mg/day appeared to have a beneficial effect on chemotherapy toxicity and 1-year survival, and progressively higher equivalent doses showed more encouraging responses (Ramos et al., 2023[158]). Therefore, in future clinical research, melatonin scheduling should be carefully tailored to dose-time ranges in which the indolamine helps control cancer comorbidity without causing toxicity. Nevertheless, the possibility of a hormesis-type biphasic response cannot be excluded, as they are common in many melatonin-coordinated adaptations to stress (Schirrmacher, 2021[176]). In this regard, to avoid side effects or cross-reactions with other drugs (Foley and Steel, 2019[52]), the active ranges and safety margins of melatonin should be established according to *i)* the most favorable phases of the circadian cycle (chronoadministration) (Ozturk et al., 2017[144]) and *ii)* the pharmacokinetic profile of each patient (Money et al., 2022[130]). Indeed, the chronobiotic facet of melatonin may open up new therapeutic approaches for oral cancer patients. For example, the circadian secretion of melatonin has been found to be associated with the improvement of radiotherapy-related side effects (Rakici et al., 2019[157]). Similarly, cisplatin-induced DNA damage can be moderated by scheduling melatonin administration to coincide with the minimum circadian expression of the *PER2* clock gene (Redondo et al., 2021[163]). Considering this *statu quo*, further *in vitro* and animal studies, as well as long-term, high-coverage, multicenter clinical trials, are needed to demonstrate the “lab-to-bedside” potential of high-dose melatonin and the feasibility of its short-term clinical application (Kim et al., 2022[84]; Menczel Schrire et al., 2022[123]). 

The translational interest of melatonin as an adjunct to first-line radiochemotherapy in cancer therapy stems from *in vitro* and *in vivo* data suggesting direct and indirect nitrooxidative protection, immunosurveillance, proapoptotic, antiproliferative, antiangiogenic and antimetastatic effects, cell cycle control and remodeling of the tumor microenvironment (Budi and Farhood, 2023[18]; Li et al., 2017[98]; Mu and Najafi, 2021[134]; Wang et al., 2022[205]; Yeh et al., 2017[219]). Accordingly, melatonin is considered to be a cytoprotector capable of activating signaling survival pathways such as Akt/mTOR and ERK/Akt (Tamtaji et al., 2019[195]) and raising barriers against proliferation, malignant progression, neovascularization, and oral cancer metastasis (Cutando et al., 2014[34]; Talib et al., 2021[194]). In this sense, some studies have reported decreased urinary (Luo et al., 2022[115]), salivary (Salaric et al., 2021[171]) and serum melatonin levels (Stanciu et al., 2020[187]; Tsai et al., 2021[199]) in oral cancer patients. Similarly, reduced antioxidant defences (Nuszkiewicz et al., 2021[139]), eventually associated with upregulated matrix metalloproteinases (mainly, MMP-9) and/or histopathological features (Luo et al., 2022[115]; Stanciu et al., 2020[186][187]), have also been reported. The antitumor protection conferred by melatonin can be observed in particular after the interruption of nocturnal secretion by ALAN (artificial light at night), which induces changes in the methylation profile associated with chronodisruption in both diurnal and nocturnal animal models (Haim et al., 2019[63]). Disruption of melatonin release in pinealectomized rats resulted in larger and thicker tongue tumors compared to sham-operated controls; also, with greater infiltration of tumor-associated macrophages and eosinophils at the invasive margin and increased expression of ERK1/2 and p53 (Kayahara et al., 2020[82]). Consistent with these observations, tongue squamous cell carcinoma SCC-15 cells treated with melatonin reduced their migration/invasion rates and aborted the activation of carcinogenic tumor-infiltrating macrophages by inhibiting the proinflammatory MIF/NLRP3/IL-β axis (Wang et al., 2023[207]). This oncostatic capacity was then reliably confirmed in melatonin-treated mice by decreased weight, volume, and MIF/NLRP3/IL-β levels in xenografted oral tumors from subcutaneous injection of SCC-15 cells (Wang et al., 2023[207]). Furthermore, SCC-25 and CAL27 oral tumor lines treated with millimolar melatonin arrested epithelial-mesenchymal transition by inhibiting the immune checkpoint PD‐L1 protein and the ERK1/2/FOSL1 pathway, and synergistically enhanced anti-PD-1 immunotherapy in a syngeneic mouse model (Luo et al., 2022[115]). 

Of relevance to cancer therapy is the drug sensitization achieved by pharmacological doses of melatonin, which ameliorated doxorubicin-induced apoptosis of skin, lung and laryngeal cancer cells compared to equivalent doses of the chemotherapeutic agent in the absence of melatonin (Fic et al., 2007[48]; Hamed et al., 2023[64]). Co-treatment with melatonin also increased the *in *v*itro*/*in vivo* cytotoxicity of 5-fluorouracil by inhibiting ERK and Akt signaling in esophageal squamous cell carcinoma cells and a mouse model (Lu et al., 2016[113]). Similarly, millimolar concentrations of the indolamine in combination with the antiangiogenic verteporfin (Wang et al., 2023[209]) downregulated the expression of epithelial-mesenchymal transition and metastasis markers in ROS-low human squamous cell carcinoma cells and in 3D-sphere cultures; impaired mitochondrial function reduced the characteristics of cancer stem cell and promoted apoptosis (Shin et al., 2022[183]). 

Mitochondria actively synthesize melatonin and are also essential for the biochemical integration and therapeutic success of exogenously administered doses (Guerra and Devesa, 2021[59]). In the context of oral cancer, the metabolic phenotype of mitochondria shifts towards hyperactive oxidative phosphorylation and ROS hyperproduction, resulting in increased mitophagy and apoptosis and reduced tumor cell proliferation (Guerra-Librero et al., 2021[60]). Thus, in CAL27 and SCC-9 oral squamous cell carcinoma cells and in CAL27xenografted mice, 0.5-1 mM melatonin induced ROS overproduction and apoptosis by uncoupling oxidative phosphorylation via reverse electron transport (Florido et al., 2022[51]). Similarly, high-dose melatonin (2 mM) combined with the ferroptosis inducer erastin achieved synergistic antitumor efficacy in human tongue squamous cell carcinoma SCC-15 cells and BALB/c nude mice xenograft by overproduction of ROS, inducing apoptosis and Fe-dependent cell death (Wang et al., 2023[206]). Similarly, 1 mM melatonin potentiated apoptosis on rapamycin-induced oral squamous cell carcinoma CAL27 and SCC-9 cells by enhancing mitochondrial ROS generation and inhibiting Akt/mTOR signaling (Shen et al., 2018[180]). However, melatonin reduced ROS generation in ROS-rich oral cancer cells through inactivation of ROS-dependent Akt and ERK signaling pathways, impairing their proliferative capacity and resistance to apoptosis (Liu et al., 2018[106]). Notably, melatonin can antagonize DNA damage and genotoxicity (Chuerduangphui et al., 2018[29]) induced by the major mutagen of the areca nut, arecoline, thus demonstrating the potential to prevent oral cancer in betel quid chewers (Shih et al., 2021[182]). In this regard, the stimulation of MMP-9 secretion by areca nut extracts in gingival keratinocytes and SAS cancer epithelial cells was attenuated by melatonin at 100 and 250 µg/mL (Chang et al., 2019[23]). In tumor-supporting neutrophils associated with oral squamous cell carcinoma cells, melatonin-dependent inactivation of the p38 MAPK and PI3K/Akt pathways aborted the release of MMP-9 and inflammatory factors, thereby reducing migration and resistance to apoptosis (Lu et al., 2017[110]). 

## The Potential of Melatonin in the Epigenetic Treatment of Patients with Oral Cancer

The pleiotropy of melatonin includes the ability to modulate gene expression and epigenetic machinery, thereby increasing the number of potential druggable targets and treatment options in human disease, including oral cancer and other neoplasms (Chuffa et al., 2020[30]; Gurunathan et al., 2021[62]; Monayo and Liu, 2022[129]). The genetic system that coordinates melatonin activity undergoes profound changes during oral carcinogenesis. For example, integration of microarray data and RNA sequencing of 12 melatonergic genes in 11 tumor types has revealed that all those with differential expression in oral cancer are consistently downregulated (Zou et al., 2021[228]). Furthermore, multivariate analysis has shown that functional loss of the *MT1* (*MTNR1A*) gene is an independent risk factor for poor prognosis and is significantly associated with tumor size and reduced overall survival in oral cancer patients (Nakamura et al., 2008[136]). On the other hand, a 3-SNP haplotype of the *MT1* promoter establishes a synergistic interplay with certain environmental factors in the promotion of oral cancer (Lin et al., 2015[100]). Further experimental evidence from oral squamous cell carcinoma SCC-9 cells showed that 1 mM melatonin inhibited the pro-angiogenic genes *HIF-1α* and *VEGF* and the pro-metastatic gene *ROCK-1* (Goncalves et al., 2014[55]).

Dysregulation of circadian genes also reveals the interplay between melatonin signaling and oral carcinogenesis (Rahman et al., 2019[156]; Rodriguez-Santana et al., 2023[167]). Indeed, knockdown of the clock-related *PER1* gene stimulated proliferation, invasion, and resistance to apoptosis of squamous cell carcinoma SCC-15 cells and increased the cellular index and volume of xenografts induced after injection inti BALB/c nu/nu nude mice (Li et al., 2016[93]). Similarly, *PER2* expression was downregulated in oral squamous cell carcinoma SCC15 and CAL27 cells, while overexpression promoted apoptosis and autophagy and inhibited proliferation (Liu et al., 2020[104]). Moreover, upregulation of the clock gene *Timeless* in human nasopharyngeal carcinoma cells is an independent prognostic factor associated with cisplatin resistance and reduced overall survival (Liu et al., 2017[107]).

The genetic dimension of melatonin expands the limited options available to treat oronasopharyngeal tumors and may mitigate their tendency to become resistant to radiochemotherapy (Russo et al., 2018[169]). In this context, melatonin is increasingly emerging as an active epigenetic actor (Capote-Moreno et al., 2019[20]; Guerra and Devesa, 2021[59]). Despite this, the epigenetic mediation exerted by melatonin in oral cancer is largely unexplored, with the sparse ongoing research mainly focused on *in vitro* and preclinical studies (Table 1(Tab. 1); References in Table 1: Ho et al., 2016[69]; Hsieh et al., 2020[71]; Hunsaker et al., 2019[73]; Li et al., 2020[94]; Nakamura et al., 2008[136]; Su et al., 2021[189]; Wang et al., 2020[210]; Yang et al., 2017[214]; Yeh et al., 2016[218]). In this complex scenario, we have updated the most recent evidence-based epigenetic achievements of the indolamine in oral cancer in the following sections. We hope that our contribution will emphasize the need for comprehensive clinical programs that convincingly demonstrate the leap forward that can be achieved by incorporating melatonin in multimodal settings for the treatment of oral cancer.

## Clinical Potential of Methylome Screening in Oral Cancer

DNA methylation is the best studied and most well-known epigenetic mark for the control of gene expression, which in mammals is essentially restricted to CpG dinucleotide cytosines, including CpG promoter islands at 5'-ends, intergenic repeat sequences and other repetitive non-coding regions scattered throughout the genome (Gazdzicka et al., 2020[54]; Moore et al., 2013[131]). Methylation is catalyzed by the DNA methyltransferase family, consisting of the conserved cytosine methylases DNMT3L, DNMT1, DNMT3A, and DNMT3B, resulting in tissue-specific maintenance methylation (by DNMT1) and *de novo* methylation patterns (by DNMT3A/B). DNA-methylated sites are then recognized by methyl-binding proteins that recruit the remodeling machinery responsible for chromatin condensation and transcriptional silencing downstream of the methylation hubs (Buitrago et al., 2021[19]). Methylation is essential for embryogenesis and for genome stability, but it is also a determinant of cancer when aberrant DNMT expression and/or activity distorts the methylation pattern of genes critical for homeostasis (Jin and Robertson, 2013[79]). In this context, methylation damage in cancer can take the form of tumor suppressors being silenced by extensive promoter methylation or, *sensu contrario*, the activation of proto-oncogenes by global hypomethylation (Kulis and Esteller, 2010[86]). Both site-specific hypermethylation and genome-wide hypomethylation have been reported to affect promoter regions (Irimie et al., 2018[75]), in both cases leading to genetic instability and mutagenicity, thus contributing to malignant progression (Nishiyama and Nakanishi, 2021[138]). Although promoter methylation is low in the canonical genome, the study of cancer-associated genome-wide hypomethylation is poorly defined compared to the suppression of critical genes by focal hypermethylation. In most cancers, hypomethylation primarily affects extragenic regions such as repetitive DNA (e.g., LINE-1 repeats), intragenic regions, transposons or intronic CpG motifs, leading to imprinting aberrations, chromosome structure abnormalities and/or activation of latent viruses and prometastatic genes (Castilho et al., 2017[21]). Either way, it is necessary to reconcile the apparent paradox of extensive promoter methylation, global genomic hypomethylation, and DNA methyltransferase hyperactivity coexisting in tumor cells, perhaps independently contributing to carcinogenesis by different mechanisms. Another intriguing issue to be elucidated is the link between epigenetic drift towards hypermethylation of promoter CpG islands in the elderly and increased susceptibility to cancer (Yu et al., 2020[222]). Given the prevalence of oral cancer in the elderly, it will be interesting to see the results of the ongoing interventional clinical trial NCT04631341 (10,000 participants, China) on the effect of melatonin on cardiovascular disease and cancer incidence in 60-74 year olds. It would also be desirable to know the results of the interventional phase 3 trial on quality of life improvement in >70 patients (123 participants enrolled) after receiving adjuvant melatonin in combination with treatment for advanced metastatic cancer (NCT02454855, France) and the multicenter, double-blind, placebo-controlled phase III trial to assess the efficacy and safety of melatonin in postoperative delirium in cancer patients over 65 (jRCTs031210673, Japan).

Accumulating evidence suggests that methylation defects are early events in cancer, strongly influenced by environmental variables and with a great capacity to determine the malignant transition (Morandi et al., 2017[132]). In this context, *ad hoc* modulation of methylation status could make epigenetics a cornerstone of cancer therapy. Chronic IL-6-mediated inflammation is a known cancer risk in the oral mucosa, disrupting normomethylation by activating the DNMT3B enzyme (Chen et al., 2014[27]), altering LINE1 hypomethylating and hypermethylating tumor suppressor genes (Gasche et al., 2011[53]). Thus, as in other neoplasms, the translational interest of the oral cancer methylome is the detection of cancer driver genes to have clinically useful diagnostic/prognostic biomarkers and/or therapeutic targets (Davoodvandi et al., 2022[36]; Demokan and Dalay, 2011[38]; Towle et al., 2013[197]; Zhou et al., 2018[227]). However, these two strategic objectives are difficult to achieve because oral cancer comprises histologically and pathologically diverse entities. Therefore, variability due to anatomical subsites, ethnicity, sex, HPV status, or environmental etiology makes tumor-specific methylation changes and/or patient individuality conceivable, as recent integrative studies have shown. Accordingly, a large number of methylation profiles will need to be characterized to understand the specificities of each tumor subcategory and stage. The popularization of mass sequencing capabilities has allowed genomic analyses to evolve from individual marker analyses to complete (epi)genome mapping. For this reason, methylation signatures are the preferred mechanism-based strategy for assessing the genetic background of tumors or the census of (epi)genetic changes resulting from environmental stressors. In addition, the recent introduction of methods to perform inexpensive assays from non-invasive liquid samples such as blood or saliva portends the reliability of genomic methylation screening tests for individual risk prediction, diagnosis and/or prognosis assessment, or the implementation of targeted therapies (Dharmawardana et al., 2020[39]; Rapado-Gonzalez et al., 2021[161], 2023[159]; Salaric et al., 2021[171]). To this end, an epigenomic and transcriptomic genome-wide screen of gingivo-buccal oral squamous cell carcinoma samples and paired healthy samples reported 209 methylation-related changes in gene expression, including important transcription factors, RNA-binding proteins, immune checkpoints, and metabolic and signaling mediators (Das et al., 2019[35]). Similarly, a recent analysis of 850,000 genome-wide methylation sites in early-stage tongue squamous cell carcinoma (TSCC), an aggressive subtype of oral cancer prone to cervical node metastasis, found methylation variations in more than 25,800 CpGs; ~79 % hypomethylated *vs*. ~21 % hypermethylated (Rapado-Gonzalez et al., 2022[160]). Notably, the hypermethylation hotspots were restricted to promoter CG-rich sequences, whereas hypomethylated CG-sequences were primarily located in the open sea region. In turn, hypermethylated CpG sequences corresponded to ~800 differentially methylated TSCC genes compared to adjacent non-tumor tissue. Comparative analysis with The Cancer Genome Atlas (TCGA) database allowed differential methylation to be narrowed down to 11 epigenetically repressed genes that showed diagnostic accuracy and therefore promise for clinical utility. As a proof of concept, 6 of them demonstrated high sensitivity and specificity in non-invasive prediction of TSCC in salivary cell pellets (Rapado-Gonzalez et al., 2022[160]). 

The pleiotropy of melatonin includes (epi)genetic modulation and can therefore influence the methylation pattern of genes relevant for carcinogenesis (Linowiecka et al., 2023[102]). The link between the indolamine and the methylation status is particularly clear in the alteration of melatonin activity by ALAN. ALAN induces dose- and wavelength-dependent global hypomethylation in cancer and healthy tissues, which has been increasingly associated with melatonin suppression. For example, in rats transferred from a 16-h dark confinement to an ALAN photoperiod with two 30-min light periods, urinary 6-sulfatoxymelatonin decreased, while DNA hypomethylation increased in a tissue-specific manner (Yonis et al., 2019[220]). Accordingly, melatonin administration could counteract the metabolic and hormonal disturbances caused by ALAN, and reduce an epigenetic trait associated with cancer incidence. Indeed, melatonin depletion and DNA methylation have been proposed as early breast cancer biomarkers (Zubidat et al., 2018[229]). Based on available evidence from several model systems, ALAN-dependent promoter methylation of the most abundant melatonin gene, *MT1,* appears to be the cause of reduced melatonin activity (He et al., 2023[67]). Consequently, *MT1* has tumor suppressor properties. In oral squamous cell carcinoma lines,* MT1* has been reported to be frequently silenced by promoter CpG island methylation, highlighting the MT1 receptor as one of the inactivation targets for oral carcinogenesis (Nakamura et al., 2008[136]). Similarly, in primary oral squamous cell carcinoma tissue samples, MT1 expression was inversely correlated with clinicopathological features of poor outcome such as tumor size or overall survival. Notably, the reactivation of melatonergic signaling by forced MT1 expression through treatment with the DNMT1 inhibitor 5-aza-2'-deoxycytidine suppressed the growth of oral squamous cell carcinoma lines (Nakamura et al., 2008[136]).

The inactivation of tumor suppressors by promoter hypermethylation may ultimately result from the upregulation of some DNA methyltransferases. In oral cancer, recent studies have reported specific increases in the three active DNMTs, DNMT1 and DNMTA/B, which are thought to be responsible for the functional silencing of numerous tumor suppressors linked to key processes of the tumor cycle such as growth, cell division, signalling, apoptosis, or adhesion (Flausino et al., 2021[49]). Thus, it is feasible to combine radiochemotherapy with specific DNMT inhibitors to restore the natural expression of overexpressed DNMT and achieve greater drug efficacy to avoid treatment resistance (Flausino et al., 2021[49]; Linowiecka et al., 2023[102]). In this regard, although some inhibitors are used as cytostatics and have been tentatively postulated for oral cancer patients (Zhou et al., 2018[227]), *in vitro* studies call for caution as they appear to reduce the efficacy of chemotherapy (Flausino et al., 2021[49]). Nevertheless, pre-treatment of esophageal cancer cells with the non-nucleoside analogue inhibitor of human DNMT1, N-phthaloyl-L-tryptophan (RG108), elicited an *in vitro* and *in vivo* transcriptomic response in 121 genes (45 upregulated *vs*. 76 downregulated), leading to increased X-ray sensitivity, apoptosis, and cell cycle arrest in the G2/M phase (Ou et al., 2018[143]). DNA demethylation appears to be involved in human dental pulp cell differentiation (Li et al., 2018[96], 2020[94]) and thus RG108 also promotes bone mineralization and dental regeneration (Sun et al., 2019[191]). Interestingly, melatonin has osteogenic, chondrogenic and osteoconductive activities (Permuy et al., 2017[153]; Wang et al., 2019[202]), which are achieved through DNMT1, MeCP2, and methylome modulation (Li et al., 2020[94]; Liu et al., 2017[105]). Therefore, RG108 may synergistically potentiate the odontogenic capacity of melatonin.

The effect on methylation of other environmental stressors, such as pollutants, is also reflected in the drop in peak serum melatonin, as occurs in humans and mice exposed to third-hand smoke (THS) (Jiang et al., 2021[78]). However, the evidence does not clarify whether the decrease is due to the effect of THS or to the protection provided by endogenous melatonin against the overproduction of THS-induced free radicals. The study by Jiang and colleagues found up to 820 genome sites with differential methylation (429 hypermethylated *vs*. 391 hypomethylated) in a human cohort of 1862 participants, including 28 THS-exposed individuals and 28 controls. Notably, the weighted gene correlation network analysis (WGCNA) performed to detect the correlation between differential methylation and the serum melatonin pool indicated that aberrant methylation of the *CYP1A2* xenobiotic metabolizer promoter is the putative driver of melatonin catabolism. Therefore, the mechanistic correlation between THS toxins and xenobiotic damage would depend on the overconsumption of endogenous melatonin scavenging activity due to the oxidative stress from THS (Jiang et al., 2021[78]). It can then be deduced that those external harmful agents are the most likely to seal epigenetic profiles with characteristic cell/tissue tropisms. From this point of view, the different tumors should be investigated for the epigenetic impact of their external risk factors. However, despite its important environmental etiology, the methylation footprint of oral cancer remains largely unexplored and is one of the strategic objectives for the coming years to improve patient prognosis and outcome. This goal is of particular interest to melatonin researchers, as undermining the indolamine's protection is the gateway to damage that can ultimately lead to cancer. New evidence in this vein was recently provided by a comprehensive multi-omic study designed to explore the role of melatonin regulators (biosynthetic enzymes, high-affinity receptors, metabolic modulators and clock genes) in 33 solid cancers (Zhang et al., 2021[225]). Based on genomic profiling and differential expression analyses of 198 and 520 esophageal carcinoma and head-and-neck squamous cell carcinoma (HNSC) samples from The Cancer Genome Atlas and Cancer Cell Line Encyclopedia, upregulation of GPR50, which inhibits the MT1 receptor through heterodimerization, was found to be significantly associated with poor survival. Furthermore, downregulation of melatonin regulators, the P450-associated enzymes CYP1A2 and CYP2C19 (in HSNC), and clock genes (*PER2* and *PER3* in HNSC and *CLOCK* in esophageal carcinoma) were also statistically associated with decreased survival. In conclusion, the relevance of oral cancer incidence to epigenetic alterations promoted by environmental aggressors and the epigenetic countermeasures employed by melatonin deserve more attention.

## Chromatin Rearrangement by Histone Modification in Oral Cancer

Histones can undergo various reversible enzyme-dependent post-translational modifications at their N-terminus, mainly acetylation, methylation, phosphorylation and ubiquitylation (Khan et al., 2015[83]). For example, acetylation is dynamically modulated by histone acetyltransferases (HATs) and histone deacetylases (HDACs). HATs, HDACs and HMTs (histone methyltransferases) are jointly responsible for the most common histone modifications observed in cancer cells. The array of acetylation and methylation tags, the so-called histone code, is critical in modulating chromatin packaging and transcription rates, either by inducing chromatin uncoiling (transcriptional activation) or condensation (gene silencing) (Le et al., 2014[90]). Acetylation removes cations from modified lysine residues and weakens histone binding to anionic DNA, making chromatin more accessible for transcription (euchromatin) and DNA less prone to methylation. Conversely, deacetylation reverts chromatin to the condensed and transcriptionally inactive configuration (heterochromatin). In addition, histone methylation can activate or repress DNA transcription, depending on the residues involved and their degree of methylation (Ren et al., 2022[165]). The disturbance of the histone code leads to genome instability and deregulation of the genetic program that maintains cell/tissue homeostasis. In this regard, there is ample evidence that abnormalities in the pattern of histone modifications cause dyshomeostasis and are often involved in oral carcinogenesis, determining tumor progression and response to treatment (Chen et al., 2013[28]; Le et al., 2014[90]; Yang et al., 2020[215]). On the other hand, there is cross-regulation between the machinery responsible for introducing histone marks and the DNA methylation/demethylation mechanisms, as remodeling of the chromatin backbone is a prerequisite for promoter opening to gain DNA accessibility (Hardeland, 2019[66]; Li et al., 2021[97]).

A recent *in silico* study of 523 oral cancer patients from The Cancer Genome Atlas database found that HDAC1 and HDAC2 enzymes were overexpressed in tumor specimens, whereas HATs showed no differences between normal and tumor tissue (Sajnani et al., 2021[170]). Indeed, previous results indicated an upregulation of HDAC2 in premalignant and oral cancer tissues and an association with tumor differentiation and TNM stages (Krishna et al., 2020[85]). However, immunohistochemical analysis of oral cancer samples provided a different perspective, as H3 hyperacetylation was significantly associated with cervical lymphatic metastasis and local recurrence (Sant'Ana et al., 2020[173]), as well as H3 trimethylation, alone or in conjunction with hyperacetylation, correlated with reduced patient survival (Shahhosseini et al., 2023[179]). Other lysine positions on histone H3 and histone H4 are also amenable to acetylation, driving effects on cell proliferation and migration, thus providing new treatment opportunities to improve patient outcomes (Li et al., 2023[95]; Lu et al., 2023[112]). Similarly, upregulation of the histone lysine methyltransferase SMYD3 has also been reported to correlate significantly with poor prognosis of oral tumors and elevated H3 trimethylation (Yang et al., 2023[216]).

By acting on reversible histone acetylation/deacetylation, melatonin modulates transcriptional competence of chromatin. The link to this epigenetic control is that the indolamine can indirectly modulate the transcriptional activity of the RZR/ROR receptor and thus ROR target genes (Ma et al., 2021[116]; Slominski et al., 2016[185]), including those related to chromatin compaction/decompaction (Jetten, 2009[77]). Melatonin has demonstrated antimetastatic activity in HSC-3 and OECM-1 oral squamous cell carcinoma cells through transcriptional suppression of the *MMP-9* gene and inhibition of its enzymatic activity (Yeh et al., 2016[218]). Critically, melatonin in the 0.5-1 mM range reduced the MAPK-ERK1/2 signaling pathway by downregulating the transcriptional coactivators CREBBP and EP300, which are required for histone acetylation at the *MMP-9* promoter and subsequent gene expression. Similar doses of melatonin also suppressed the expression and activity of the MMP-9 protein, thereby inhibiting the migration and invasion of the human nasopharyngeal carcinoma cell lines HONE- 1 and NPC-39 (Ho et al., 2016[69]). On this occasion, however, melatonin suppressed the *MMP-*9 promoter-binding activity of the transcription factor SP-1 by inhibiting the JNK/MAPK pathway. 

Nuclear histone lysine-specific demethylases (LSDs) remove methyl groups at specific lysine-histone positions, which can lead to gene repression or stimulation . In oral cancer, LSDs have recently been suggested as promising therapeutic targets. Indeed, histone lysine-specific demethylase 1 (LSD1) was found to be inadequately upregulated in oral squamous cell carcinoma tissue, where it correlated with tumor stage, and in an orthotopic oral cancer mouse model, where LSD1 overexpression was found to be metastagenic, whereas LSD1-knockdown abrogated tumor spread (Alsaqer et al., 2017[8]). Therefore, LSD1 activation appears to be essential for tumor growth and metastatic spread of oral tumors and therefore a potential new diagnostic biomarker and therapeutic target. In this context, aberrant overexpression of LSD1 correlated with poor prognosis in a cohort of 78 oral cancer patients (Yang et al., 2017[214]). Notably, pharmacological melatonin has been shown to significantly downregulate LSD1 in LSD1-overexpressing oral cancer patient-derived tumor xenografts and oral cancer cell lines, in addition to arresting the cell cycle at the G0/G1 phase and decreasing cell proliferation in a dose- and time-dependent manner. At the same time, H3 acetylation increased at the H3K4 and H3K9 sites (Yang et al., 2017[214]). 

Therefore, the epigenetic regulation of the non-coding transcriptome by melatonin signaling in normophysiological and pathological contexts opens new approaches for the treatment of oral cancer. A pro-metastatic nuclear lncRNA, melatonin-regulated oral cancer stimulator (MROS-1), binds to the *de novo* methylation enzyme DNMT3A (DNA methyltransferase 3A) to promote oral cancer cell migration by suppressing the expression of the tumor suppressor prune homolog 2 (PRUNE-2) and the activation of the JAK-STAT pathway (Su et al., 2021[189]). In human oral squamous cell carcinoma cells (OECM-1, HSC-3 and HSC-4), melatonin (1 mM) inhibited TPA (12-O-tetradecanoylphorbol-13-acetate)-induced migration by downregulating the metastatic potential of the procarcinogen MROS-1, which antagonizes PRUNE-2 (Su et al., 2021[189]).

## The Role of microRNAs in the Etiopathogenesis and Treatment of Oral Cancer

MicroRNAs (miRs) are conserved small (21-23 nt) non-coding RNAs involved in the regulation of gene translation of up to 60 % of human protein-coding genes. By hybridizing to the 3'-UTR tail of targeted mRNAs, miRs determine their subsequent post-transcriptional course towards translational inhibition or degradation (O'Brien et al., 2018[140]). The most recent estimate is that there are approximately 2,600 mature miRs present in the human genome (miRBase, v.29). In addition to their significant number, each miR can simultaneously target many different mRNAs, and each mRNA can be targeted by a variety of miRs, either simultaneously or separately under specific regulatory conditions or cell types. Epigenetic biases of the miR-mRNA interactome have profound consequences, including malignant transformation and traits of the tumor phenotype, such as uncontrolled growth, resistance to apoptosis, immune evasion, angiogenesis, or invasion (Peng and Croce, 2016[151]). Thus, the growing evidence that the aberrant expression of miR genes, due to alterations in the methylation of their CpG islands, becomes cardinal for the implantation and propagation of human tumors, makes it necessary to update the models of cancer etiopathogenesis. Accordingly, modulation of oncomiRs or oncosuppressor miRs is an open avenue for novel miR-based therapies. In line with this perspective, miRNA signatures vary significantly during malignant transformation and are thought to be specific for different tumors and stages (Wang et al., 2021[203]). Therefore, miR mapping provides myriad candidates for diagnostic, progression and follow-up markers, as well as potential druggable therapeutic targets (Lubov et al., 2017[114]; Tucci, 2022[200]).

The importance of these new players in oral cancer is relevant because miRs are present intracellularly and extracellularly, most of them also in body fluids such as saliva, oral swirls, or blood, facilitating their high-quality profiling (Lan et al., 2015[88]; Patel et al., 2011[147]) to detect tumor heterogeneity (Muthu and Narayanan, 2021[135]). In this context, a systematic review from the interim 2008-2020 investigated the feasibility of salivary miRs as biomarkers for the early detection of oral cancer and found 13 downregulated and 12 upregulated species with diagnostic or prognostic potential (Al Rawi et al., 2021[5]). Only four of them appeared in more than one of the reports, demonstrating the heterogeneity of the findings. In a more recent systematic review published with data up to July 2022, three salivary miRs showed discriminatory potential for recurrence and malignant progression of oral diseases: miR-21, miR-31 and miR-184 (Liu et al., 2023[108]). Furthermore, the design of miR panels as discriminatory algorithms for high- or low-risk tumors gives these small gene regulators enormous potential in clinical epidemiology and evidence-based oral cancer treatment. Thus, five dysregulated miRs, including miR-21, present in oral swirls of patients with squamous cell carcinoma were able to screen high-risk cancer patients and specifically discriminate oral cancer from other potentially malignant phenotypes (Yap et al., 2019[217]). The enormous potential of these panels can be appreciated in some recent studies of miRs associated with oral cancer risk factors (Aghiorghiesei et al., 2022[4]) or specific populations, such as Ecuadorian mestizos with oral cancer (Salazar-Ruales et al., 2018[172]). In the last of these papers, four population-selective miR species showed overexpressed levels in a cohort of more than one hundred patients and controls, as well as a distinct association with primary tumor location, TNM stages or HPV infection.

The popularization of next-generation sequencing is increasing the reliability of miRnome pictures from different sub-sites or stages of malignancy. In this regard, some large-scale meta-analyses reporting miR collections with well-defined alterations in patients with different subtypes of oral cancer are raising the hope of identifying prognostic biomarkers to improve treatment plans and patient outcomes (Rishabh et al., 2021[166]). Indeed, a systematic review and a subsequent meta-analysis up to 2017 recorded 27 increases and 26 decreases in miRs associated with a poor prognosis, highlighting the overexpression of miR-23 in cases of reduced survival (Lubov et al., 2017[114]). Similarly, Wang and colleagues recently investigated 35 miRs that have been reported to be differentially expressed in oral cancer over the past 5 years (Wang et al., 2021[203]). Subsequently, the possibility of targeting specific miRNAs with inhibitors (anti-miR oligonucleotides or miR sponges) or enhancers (miR mimics or agomiRs) is providing new insights to specifically counteract their cancer-induced changes and override the tumor phenotype (Min et al., 2015[126]; You et al., 2020[221]). In particular, transfection of human oral squamous cell carcinoma SCC-4, SCC-9 and SCC-25 cells with miR-29a, which is downregulated in oral squamous cell carcinoma tissue, directly targeted the *MMP2* gene and reduced its tumor-associated overexpression, thereby attenuating the apoptotic and invasive potential of oral tumor lines (Lu et al., 2014[111]). In addition, miRs may provide synergistic efficacy in combination with conventional first-line chemotherapy for oral cancer: cisplatin, 5-fluorouracil, and paclitaxel (Meng et al., 2021[124]). Nevertheless, miR expression also changes in response to chemotherapy, reflecting their involvement in drug sensitization and the possibility of manipulating the miR functional network to predict and control treatment efficacy. It should be noted that miRs cross gap junctions and are usually present in extracellular vesicles, thus providing a vehicle to enhance metastatic potential in the tumor microenvironment, as well as chemosensitization and chemoresistance in neighboring cells (Yamaguchi et al., 2022[213]). In a proof-of-concept study, Huni and colleagues recently documented the regulatory pathways by which miR-375 expression and miR-21 downregulation operate in the triple-resistant (cisplatin, 5-fluorouracil, and taxol) oral squamous cell carcinoma lines SCC-9 and SCC-25 (Huni et al., 2023[72]).

In conclusion, miR compilations from research platforms, such as oral tumor lines, biological fluids or tissue samples offer promising solutions for the development of diagnostic/prognostic biomarkers and/or targeted therapies. However, the amplitude and heterogeneity of published miR-alteration datasets suggest difficulties prior to clinical translation. In a recent review, Rishabh et al. (2021[166]) reported 117 miRs involved in oral tumor initiation and development, and a further 67 species with diagnostic potential, including 20 with probable pathogenic involvement (Rishabh et al., 2021[166]). In addition to high heterogeneity, miRnomes showed inconsistencies due to inhomogeneity in cohort size, stratification (by ethnicity, age, and sex), and technical issues related to different sample processing methods, study design, or unreliability of housekeeping miR genes for normalization (Mazumder et al., 2019[122]). Therefore, further *in vitro* and animal studies are needed to achieve a reliable description of the miRnome during the tumor cycle of different oral cancer subtypes. In addition, well-planned, standardized, and randomized longitudinal large-scale clinical trials should be conducted to test the diagnostic/predictive robustness of the best miR candidates (Wang et al., 2021[203]). This arduous work, now facilitated by whole-genome sequencing facilities, could provide the silver bullets that will usher in a new era in the fight against oral cancer.

Recent efforts to unravel the melatonin-controlled miR network in oral cancer cells have hypothesized that some indolamine-regulated species may be critical in attenuating carcinogenicity and tumor severity. In this regard, a 2010-2020 meta-analysis of oral and other cancers has shown that melatonin orchestrates the upregulation of 29 miRs and the downregulation of 17 miRs, targeting 604 and 749 validated genes respectively, 90 of which are subject to dual up/down regulation (Chuffa et al., 2020[30]). Oral cancer showed the second largest miR set (after breast cancer), consisting of 118 genes controlled by overexpressed miRs and a further 208 genes under the regulatory influence of downregulated species. Two members of this plethora play a prominent role in arresting the malignant progression of oral tumors: miR-34b-5p, which targets the *ABCB1* gene encoding an ATP-dependent drug efflux pump, and the *CYP1A1* repressor miR-892a, a member of the cytochrome P450 superfamily (Hsieh et al., 2020[71]). In this sense, cells resistant to the cytostatic drug vincristine (VCR), derived from the oral tumor lines SAS and SCC-9, which characteristically express low levels of miR-34b-5p and miR-892a, significantly increased their expression due to the genetic and biochemical counterbalances induced by melatonin. In particular, 0.5-2 mM melatonin, with or without the alkaloid VCR, reduced the viability and colony-forming ability of VCR-resistant cells *in vitro* and *in vivo* (in a murine orthotopic engraftment model) by stimulating their apoptotic response through the MAPK and AKT pathways (Mihanfar et al., 2022[125]). Furthermore, melatonin increased VCR sensitivity by downregulating *ABCB1* and *ABCB4* genes *in vitro* and *in vivo*. In conclusion, melatonin-induced upregulation of miR-34b-5p and miR-892a promoted the death of VCR-resistant cells while increasing their sensitivity to VCR. Thus, the indolamine raises the status of a chemotherapeutic agent of great interest to overcome chemotherapy resistance of recurrent oral tumors (Hsieh et al., 2020[71]). 

The modulation exerted by melatonin on the miR transcriptome has shed light on some of the mechanisms by which its known anti-inflammatory effect operates. The mouse microglial cell line N9 exposed to proinflammatory lipopolysaccharide and adenosine triphosphate (LPS plus ATP) showed 136 differentially expressed miRs; cotreatment with melatonin (0.5 mM) affected 10 miR species. Notably, miR-155-3p, which was upregulated in association with (LPS plus ATP)-induced NLR family pyrin domain containing 3 (NLRP3) inflammasome, was significantly reduced by melatonin. The same response was obtained *in vivo* with melatonin doses of 30 mg/kg (4 times at 6 h intervals starting 2 h before LPS injection), which protected the brain of mice against inflammasome activation (Tarakcioglu et al., 2022[196]). The exosomes of oral cancer lines SCC-9, SCC-25, and CAL27 contain prometastatic miR-155, which is reduced by a 10 µg/mL dose of melatonin administered for 72 h to restore the serum and salivary levels achieved after ingestion of commercially available formulations (Hunsaker et al., 2019[73]). However, the same dose of melatonin increased the exosomal/oncosomal presence of miR-21, which is associated with poor prognosis of oral tumors, perhaps an epiphenomenon involving other transcriptional mediators that have binding sites in the miR-21 promoter (Hunsaker et al., 2019[73]).

Cultured SCC-9 cells exposed to millimolar melatonin for 48 h showed higher rates of apoptosis, which significantly reduced their viability and ability to proliferate, migrate and metastasize (Wang et al., 2020[210]). The attenuation of the tumour potential occurred in parallel with melatonin-dependent overexpression of miR-25-5p, which is frequently downregulated in tumour tissue. In addition, inhibition of miR-25-5p suppressed the oncostatic capacity of melatonin in SCC-9 cells (Wang et al., 2020[210]). Therefore, downstream melatonin signaling appears to control miR-25-5p in oral cancer. In addition, RT-qPCR showed that miRNA-25-5p could target the* NEDD9* gene (Wang et al., 2020[210]), which stimulates MMP-9 release and invadopodia formation, two critical steps in conditioning the extracellular matrix for cell migration and invasion (Grauzam et al., 2018[58]). It should be noted that in a randomized trial of 50 patients, a 20 mg dose of melatonin combined with neoadjuvant chemotherapy reduced the percentage of residual tumor, although it was not statistically significant, as well as the expression of hypoxamir miR-210 and stem cell marker CD44 (Kartini et al., 2020[81]), two factors that proactively contribute to the hypoxia of the microenvironment that favors chemoresistance.

There is a growing view that specialized interaction networks act on specific tumor types or at specific stages of the tumor cycle. In this regard, current technology can be instrumental in ensuring that the molecular detailing of the different gnoseological entities leads to treatments tailored to the idiosyncrasies of patients.

## Conclusions and New Perspectives

The high prevalence of cancer is a global health problem, exacerbated by those epidemiological projections of a progressive increase in new cases until 2040, when a global burden of 28.4 million is expected (Sung et al., 2021[193]). Furthermore, the toxicity associated with first-line radiochemotherapy and resistance to therapy usually lead to aggressive relapse of advanced carcinomas and poor patient outcomes (Adhikari et al., 2022[3]), especially in young people with oral cancer (Mohideen et al., 2021[128]). The urgency of making cancer chronic to reduce the mortality burden has focused the efforts of health care services, although some cancers, such as oral tumors, continue to have devastating mortality rates. To alleviate this problem, new treatment alternatives have attracted funding and cutting-edge technology on a “big science” scale over the last half century. In this scenario, melatonin has emerged as a promising oncostatic agent (Reiter et al., 2017[164]), mainly indicated for integrative approaches in which standardized first-line treatments are accompanied by interventions capable of partially alleviating their detrimental side effects. In addition, combined formulations may enhance therapeutic efficacy, avoid treatment interruptions or allow effective doses to be increased. Accordingly, recent meta-analyses of randomized controlled trials agree that adjuvant melatonin makes a positive contribution to 1-year survival in cancer patients (Lim et al., 2022[99]). The low cost and few side effects of melatonin supplementation are additional reasons to continue basic and clinical research in large cohorts to test its therapeutic scope.

The omnipresence of melatonin in the living world has provided clues to understand that its position goes beyond the stabilization of the circadian/seasonal cyclicity of the suprachiasmatic "master biological clock". Rather, the systemic functions performed by melatonin correspond to those of a cellular protector, determinant of tissue homeostasis through neutralization of redox imbalance, immunostimulation or anti-inflammatory, antiviral, and anticancer defense, among other relevant facets (Wang et al., 2022[205]). Consequently, melatonin is attracting increasing interest in cancer research, as evidenced by the volume of published papers, which has quadrupled since the beginning of this century according to PubMed. Part of this literature may be inflationary and respond to the malpractice of invoking stereotyped mantras without providing additional experimental data, as recently emphasized (Boutin et al., 2023[14]). However, the contents of the previous sections are not topics that have been cloned a thousand times but rather the results of *in vitro* and animal studies that, together with the reported beneficial effect of melatonin in the treatment of post-operative or radiochemotherapy side effects, convincingly support the antitumor potential of this extraordinary molecule.

The evidence for melatonin-mediated oncostasis is mainly functional (observational), whereas high-throughput detection techniques are increasingly revealing signaling networks, candidate genes, and epigenes targeted by the indolamine. It is worth noting that the mechanistic description of how this interactional network mediates the carcinogenic process remains to be elucidated. In particular, there is a need to validate the increasingly known melatonin-triggered genome-epigenome interactome, so that decision-making for new evidence-based treatments can take advantage of the (epi)genetic effects of the indolamine. Indeed, DNMTi leads to severe side effects that could hypothetically be alleviated by melatonin in combination treatments (Davoodvandi et al., 2022[36]; Linowiecka et al., 2023[102]). Similarly, epidrugs such as melatonin-HDACi (Helmi et al., 2023[68]) or melatonin-STAT3 hybrids (Zhang et al., 2023[224]) show potent anticancer effects. A major milestone in this agenda will be the characterization of the (epi)genetic profiles and melatonin-mediated responses associated with tumor subtypes and/or stages, which would foreseeably allow subclinical diagnosis, arrest of progression, and individualized treatments. In addition, (epi)genome-wide association screenings and candidate gene studies suggest that MT1 may mediate (epi)genome-environment relationships (Gottschalk et al., 2020[57]). This interplay hints at the prophylactic utility of melatonin as a countermeasure to control epigenetic damage from external/occupational factors that ultimately influence oral carcinogenesis (Lin et al., 2015[100]). Therefore, there is an urgent need for translational research in oral cancer, where the reparative capacity of melatonin remains largely unknown and, if confirmed, could help to overcome the current difficulties in its clinical management. 

The research focus of our team is to explore the therapeutic potential of melatonin. We have made mechanistic contributions to the redox potential of the indolamine (Parada et al., 2014[145]; Romero et al., 2010[168]), as well as *in vitro* investigations and studies in animal models (Farre-Alins et al., 2020[45]; Patiño et al., 2016[149]) indicating its translational potential. Against this background, we encourage physicians and clinical researchers to conduct cohort studies which, in the medium- to long-term, will shed light on the ability of melatonin to improve the clinical management of entities in need of substantial progress in this area, such as oral cancer.

## Notes

Emilio Gil-Martín and Alejandro Romero (Department of Pharmacology and Toxicology, Faculty of Veterinary Medicine, Complutense University of Madrid, 28040 Madrid, Spain; E-mail: manarome@ucm.es) contributed equally as corresponding author.

## Declaration

### Acknowledgments 

A.R. would like to thank UCJC (INFLAMAMEL 2022-07 project) for its continuous support. 

### Conflict of interest 

The authors declare that they have no conflict of interest. 

## Figures and Tables

**Table 1:**
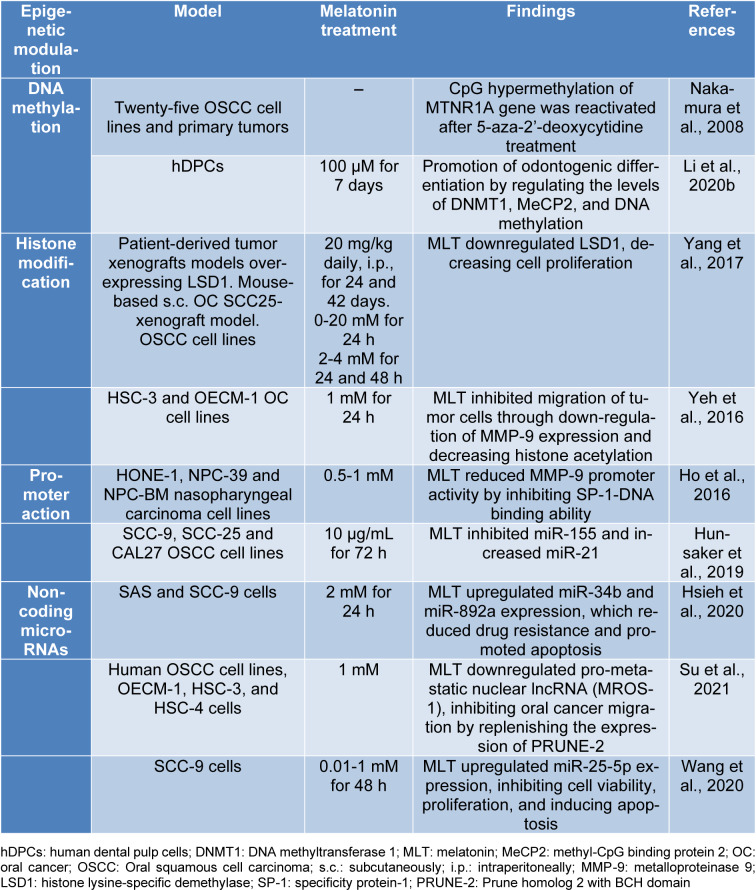
Melatonin's role on epigenetic modulation of oral cancer
